# A Supercluster of Neutralizing Epitopes at the Interface of Ricin’s Enzymatic (RTA) and Binding (RTB) Subunits

**DOI:** 10.3390/toxins9120378

**Published:** 2017-11-23

**Authors:** Amanda Y. Poon, David J. Vance, Yinghui Rong, Dylan Ehrbar, Nicholas J. Mantis

**Affiliations:** 1 Department of Biomedical Sciences, University at Albany School of Public Health, Albany, NY 12201, USA; aypoon@albany.edu; 2 Division of Infectious Disease, Wadsworth Center, New York State Department of Health, Albany, NY 12208, USA; david.vance@health.ny.gov (D.J.V.); yinghui.rong@health.ny.gov (Y.R.); dylan.ehrbar@health.ny.gov (D.E.)

**Keywords:** ricin, antibody, neutralizing, epitope

## Abstract

As part of an effort to engineer ricin antitoxins and immunotherapies, we previously produced and characterized a collection of phage-displayed, heavy chain-only antibodies (V_H_Hs) from alpacas that had been immunized with ricin antigens. In our initial screens, we identified nine V_H_Hs directed against ricin toxin’s binding subunit (RTB), but only one, JIZ-B7, had toxin-neutralizing activity. Linking JIZ-B7 to different V_H_Hs against ricin’s enzymatic subunit (RTA) resulted in several bispecific antibodies with potent toxin-neutralizing activity in vitro and in vivo. JIZ-B7 may therefore be an integral component of a future V_H_H-based neutralizing agent (VNA) for ricin toxin. In this study, we now localize, using competitive ELISA, JIZ-B7’s epitope to a region of RTB’s domain 2 sandwiched between the high-affinity galactose/N-acetylgalactosamine (Gal/GalNAc)-binding site and the boundary of a neutralizing hotspot on RTA known as cluster II. Analysis of additional RTB (*n* = 8)- and holotoxin (*n* = 4)-specific V_H_Hs from a recent series of screens identified a “supercluster” of neutralizing epitopes at the RTA-RTB interface. Among the V_H_Hs tested, toxin-neutralizing activity was most closely associated with epitope proximity to RTA, and not interference with RTB’s ability to engage Gal/GalNAc receptors. We conclude that JIZ-B7 is representative of a larger group of potent toxin-neutralizing antibodies, possibly including many described in the literature dating back several decades, that recognize tertiary and possibly quaternary epitopes located at the RTA-RTB interface and that target a region of vulnerability on ricin toxin.

## 1. Introduction

**Figure 1:**
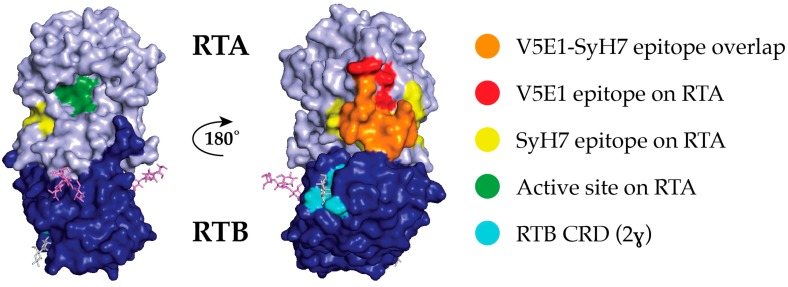
V5E1 and SyH7 epitope localized on surface depiction of ricin toxin. Ricin holotoxin (PDB ID 2AAI) displayed in PyMol showing ricin’s enzymatic subunit (RTA; light blue) and ricin toxin’s binding subunit (RTB; dark blue). The overlap between SyH7 and V5E1 epitopes is shown in orange; SyH7’s additional epitope coverage is in yellow, and V5E1’s additional epitope coverage is in red. Also highlighted are RTA’s active site (green), and RTB’s 2γ Gal/GalNAc binding pocket (sky blue) with lactose molecule (gray) (right image).

Ricin toxin, a product of the castor bean plant (*Ricinus communis*), is the archetype Type II ribosome-inactivating protein (RIP) [1]. Ricin is initially synthesized as preprotein, but accumulates in storage vesicles as a mature, 65 kDa glycosylated protein in which the two subunits, RTA and RTB, are joined by a single disulfide bond [2,3,4]. Ricin’s enzymatic subunit (RTA) is an RNA N-glycosidase (EC 3.2.2.22) that catalyzes the hydrolysis of a conserved adenine residue within the sarcin/ricin loop (SRL) of 28S rRNA [5,6,7]. It is a globular protein with 10 β-strands (a–j) and seven α-helices (A–G) [8,9]. RTA’s active site forms a shallow pocket that includes Tyr80, Tyr123, Glu177, Arg180, and Trp211 (Figure 1). RTA’s C-terminus (residues 211–267) forms a protruding element that interacts with RTB [8]. Residue Cys259 of RTA forms a disulfide bond with Cys4 of RTB.

RTB (262 residues) is a galactose (Gal)- and N-acetylgalactosamine (GalNAc)-specific lectin responsible for attachment to glycolipid and glycoproteins on the surfaces of mammalian cells, including lung epithelial cells [10,11]. Structurally, RTB consists of two globular domains with identical folding topologies [8]. Each of the two domains (1 and 2) is comprised of three homologous sub-domains (α, β, γ) that arose by gene duplication from a primordial carbohydrate recognition domain (CRD) [8,10,12]. Only the external sub-domains, 1α and 2γ, retain carbohydrate recognition activity: sub-domain 1α (residues 17–59) is specific for Gal, whereas 2γ (residues 228–262) recognizes Gal and GalNac [9,13,14]. Although RTB’s overall affinity for monosaccharides is quite low (K_D_ in the range 10^−3^ to 10^−4^ M), its affinity for complex sugars on the surface of cells is 3–4 magnitudes greater [15].

As part of an effort to engineer ricin antitoxins and immunotherapies, we have produced and characterized libraries of phage-displayed, heavy chain-only antibodies (V_H_Hs) from alpacas hyperimmunized with non-toxic mixtures of recombinant RTA, RTB, and ricin toxoid [16]. We recently described the full-length DNA sequences, binding affinities, epitope specificities, and neutralizing activities of 68 unique V_H_Hs: 31 against RTA, 33 against RTB, and four against ricin holotoxin [17]. Epitope positioning of these V_H_Hs was achieved by cross-competition ELISAs with a panel of toxin-neutralizing monoclonal antibodies (mAbs) against known (or postulated) epitopes on RTA [18,19] and RTB [20]. The 68 V_H_Hs grouped into >20 different competition bins, revealing the diversity of antibody binding sites on ricin toxin.

**Figure 2:**
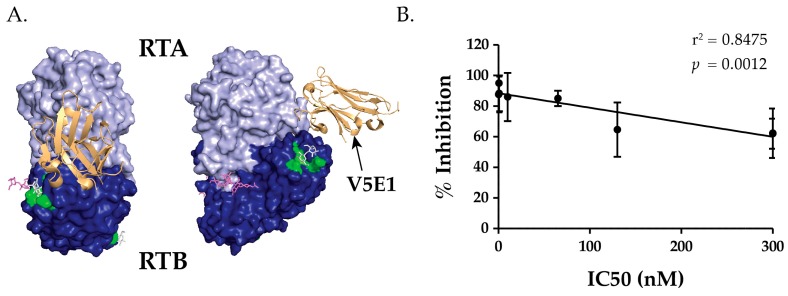
Relationship between V5E1 competition and toxin-neutralizing activity. (**A**) The crystal structure of V5E1 (gold) in complex with RTA (light blue) and superimposed on ricin holotoxin (RTB, dark blue), from [24]. RTB’s 2γ Gal/GalNAc binding pocket is colored green; (**B**) Linear regression analysis of IC_50_s of Tier I and Tier II V_H_Hs versus competition (% inhibition) with V5E1 (*n* = 8; *r*^2^ = 0.8475; *p* = 0.0012). Tier III V_H_Hs were not included in this analysis since they did not produce quantifiable IC_50s_. Each point on the graph represents a V_H_H. IC_50_ values are the mean of at least three technical replicates, while % inhibition values are the mean and SD of three technical replicates. Refer to the Material and Methods for additional details.

The RTA-specific V_H_Hs with the strongest toxin-neutralizing activities were confined to bins that overlapped with two previously known neutralizing hotspots, the so-called clusters I and II. Cluster I, defined by mAb PB10, is focused around RTA’s α-helix B (residues 98–106), a secondary element conserved among RIPs [19,21,22,23]. Cluster II, defined by mAb SyH7, centers around RTA’s α-helix F and the F-G loop situated on RTA’s “backside”, relative to the active site [19,22]. In the context of ricin holotoxin, cluster II epitopes on RTA are in close proximity to RTB domain 2. In fact, the recently described crystal structure of V_H_H V5E1 bound to RTA (PDB ID 5J57) indicates that the V5E1’s complementarity determining regions (CDR) 1 and 2 make contact with RTA, while CDR3 could likely interact with RTB in the context of ricin holotoxin (Figure 2) [24]. Thus, V5E1 recognizes a quaternary epitope that encompasses parts of RTA cluster II and RTB domain 2.

JIZ-B7 (also known as RTB-B7) is an RTB-specific V_H_H identified in our first series of pannings of the so-called HobJo alpaca library [16]. Among the original nine RTB-specific V_H_Hs that were identified, JIZ-B7 was the only one that had in vitro toxin-neutralizing activity. For that reason, it was chosen as the de facto partner to pair with different RTA-specific V_H_Hs in the design of bispecific antitoxins [16,25,26]. The resulting V_H_H heterodimers proved to be highly effective at neutralizing ricin toxin in vitro and in vivo, possibly because of their propensity to induce toxin aggregation in solution and on cell surfaces [27].

However, pinpointing JIZ-B7’s actual binding site on ricin toxin has proven difficult: JIZ-B7 did not react with an RTB peptide array, nor was it competitively inhibited from binding to ricin by any of the RTB-specific mAbs that are in our collection [16,25]. It was therefore fortuitous to discover that JIZ-B7 was competitively inhibited from binding to ricin by SyH7 [17]. As noted above, SyH7 recognizes an epitope within cluster II on the backside of RTA, in close proximity with the interface of RTB domain 2 (Figure 1). We therefore hypothesize that JIZ-B7’s epitope on RTB is located near the cluster II footprint on RTA, at the border of RTA and RTB.

In the current study, we have now tentatively localized, using competition ELISA, JIZ-B7’s epitope to RTB subdomain 2γ, which is located in close proximity to SyH7’s epitope on RTA. Moreover, we have positioned the epitopes that are recognized by an additional panel of toxin-neutralizing and non-neutralizing RTB- (*n* = 8) and holotoxin-specific (*n* = 4) V_H_Hs. Overall, the results are indicative of there being a “supercluster” of epitopes at the RTA-RTB interface with a neutralizing hotspot at or near its core. Among the V_H_Hs tested, toxin-neutralizing activity was most closely associated with epitope proximity to RTA, not interference with RTB’s ability to engage Gal/GalNAc receptors. These and other results suggest that antibody engagement with epitopes within this supercluster neutralize ricin by perturbing toxin uptake and/or intracellular trafficking, not blocking the attachment to cell surfaces.

## 2. Results

### 2.1. Characterization of RTB- and Holotoxin-Specific V_H_Hs that Compete with SyH7

As noted above, we recently discovered that JIZ-B7 was unable to bind ricin holotoxin when captured in a sandwich ELISA by SyH7 [17]. SyH7 recognizes an epitope on the backside of RTA near RTB’s domain 2 (Figure 1; Table S1), suggesting that JIZ-B7’s epitope may be in the vicinity of the RTA-RTB interface. JIZ-B7 is not unique, as we recently identified an additional 12 RTB- and holotoxin-specific V_H_Hs whose binding to ricin was also impacted negatively in a SyH7 sandwich ELISA (Table S1) [17]. By direct ELISA, nine V_H_Hs recognize RTB (JIZ-B7, as well as V5E4, V2C11, V5G1, V2D4, V4A1, V5H2, V6B9, V8D12) and four recognize ricin holotoxin, but not the individual RTA or RTB subunits (V5D1, V1B4, V5G6, V5G12). The SyH7 competition results by sandwich ELISA were confirmed using a slightly different assay, known as EPICC (see Materials and Methods). In the EPICC assay, biotinylated-ricin was incubated in solution with competitor V_H_Hs (e.g., JIZ-B7), and then applied to SyH7-coated microtiter plates and detected with streptavidin-HRP. This competition strategy (unlike the sandwich ELISA) ensured that the query V_H_Hs had access to their epitopes prior to competition with SyH7. Overall, there was good agreement between the two assays (Table S1), although the absolute degree of competition varied considerably, probably because of the large differences in the relative affinities. Two RTB-specific control V_H_Hs (V5D5, V5H6) whose ability to bind ricin was not affected by SyH7 were included in the panel. Thus, in total, we have a collection of 13 V_H_Hs that tentatively recognize epitopes at the RTA-RTB border, which we will refer to as Supercluster II (SCII).

As shown in Table 1, we next rank ordered the 13 SCII V_H_Hs, plus two control V_H_Hs, by their in vitro toxin-neutralizing activities (TNA). V_H_Hs were categorized as Tier I if their IC_50_s were <10 nM, Tier II if their IC_50_s were between 10 nM and 300 nM, and Tier III if their IC_50_s were >300 nM. V_H_H binding affinities for ricin holotoxin are also provided in Table 1. Tier I contains JIZ-B7 plus three additional V_H_Hs (V5E4, V2C11, V5G1). These four V_H_Hs are specific for RTB but are not clonally related, as evidenced by different CDR3 lengths and primary amino acid sequences (Figure S1A). Tier II also contains four V_H_Hs, with IC_50_s ranging from 65–300 nM and equilibrium dissociation constants (K_D_) between 0.19–3.2 nM. One of the Tier II V_H_Hs is specific for ricin holotoxin, and the other three for RTB. Finally, in Tier III, there are five V_H_Hs in which three are specific for ricin holotoxin and two for RTB. The Tier III V_H_Hs are, by definition, lacking in TNA (i.e., IC_50_ > 300 nM). The two control V_H_Hs (V5D5, V5H2) are specific for RTB, but have relatively poor binding affinities for ricin toxin and no detectable TNA. It is of interest to note that the CDR3s of V2C11 (Tier I), V2D4 (Tier II), and V5H2 (Tier III) are identical in length and have considerable primary amino acid sequence identity, indicating that they likely derived from the same B cell lineage (Figure S1B). In addition, in a recent report, we noted that V_H_Hs with the highest binding affinity (K_D_) generally had the strongest TNA, and that TNA was associated with a slower off rate (k_d_) rather than faster on rate (k_a_) [17]. Others have noted a similar relationship [28].

### 2.2. Epitope Positioning by V5E1 Competition

As a strategy to further refine the epitopes in Supercluster II, we performed competition assays with V5E1, an RTA-specific V_H_H that binds ricin with high affinity (0.02 nM) and has potent toxin-neutralizing activity (0.5 nM IC_50_) [24]. The X-ray crystal structure of V5E1 bound to RTA (PDB ID 5J57) indicates that V5E1’s complementarity determining regions (CDR) 1 and 2 make contact with RTA, while CDR3 likely interacts with RTB’s subdomain 2γ (Figure 2A). Microtiter plates were coated with V5E1 and then assessed for the ability to capture biotinylated ricin that was pre-incubated with competitor V_H_Hs in solution. As shown in Table 1, all 13 V_H_Hs in Tiers I-III were competed to varying degrees with V5E1, with the most notable competitor being JIZ-B7 (95%). The remaining three Tier I V_H_Hs were also potent competitors of V5E1 (86–88%), as was a single Tier II antibody, V5D1 (85%). The remaining three Tier II V_H_Hs were good competitors (62–64%), while the Tier III V_H_Hs were weak to moderate inhibitors (37–42%), with the notable exception of V1B4 (85%) (see below). Control antibodies (V5D5 and V5H6) did not compete with V5E1 (<10%).

Within the Tier I and II V_H_Hs, there was a statistically significant linear relationship between toxin-neutralizing activity (IC_50_) and competition with V5E1, as demonstrated in Figure 2B. A Pearson’s correlation coefficient of −0.9206 (*p* = 0.0012) was calculated between IC_50_ values and competition with V5E1, as well. Thus, we propose that there is a neutralizing focal point at the RTA-RTB interface, which is accessible by making primary contact with RTA (e.g., V5E1) or RTB (e.g., JIZ-B7). Holotoxin-specific antibodies like V5D1 may straddle that divide. V1B4, however, is somewhat of an anomaly in that it has a relatively weak affinity for ricin holotoxin (4 nM) and lacks TNA, even though it is as effective (or nearly as effective) as the Tier I V_H_Hs (e.g., V2C11, V5G1) at inhibiting V5E1 from capturing ricin. We speculate that V1B4 may recognize a “cold spot” within Supercluster II, although additional competition and differential binding assays are needed to localize V1B4’s epitope with real confidence.

### 2.3. Cross-Competition ELISAs Indicate That Tier I V_H_Hs Recognize Overlapping Epitopes on RTB

Based on their capacities to compete with V5E1, we hypothesized that the Tier I V_H_Hs (V5E4, JIZ-B7, V2C11, V5G1) likely recognize overlapping epitopes on RTB. To test this hypothesis, the V_H_Hs were subject to cross-competition binding assays (Table 2). As expected, each V_H_H was able to compete with itself, as evidenced by >94% reduction in the binding of the analyte. Pairwise competitions with the Tier I V_H_Hs indicated that V5E4, JIZ-B7, V2C11, and V5G1 do indeed compete with each other. However, there was non-reciprocal competition in the case of JIZ-B7. As the capture antibody, JIZ-B7 partially affected Tier I V_H_Hs from binding ricin (43–48%). However, as a competitor antibody (in solution), JIZ-B7 was able to compete with the three other Tier I V_H_Hs (94–96%). This disparity is not easily explained by binding affinity, since the Tier I antibodies have equilibrium dissociation constant (K_D_) constants ranging from 0.006 to 0.106 nM. In the case of several HIV-1 specific mAbs like PGT135, non-reciprocal competition ELISAs were attributed to the induction of conformational/allosteric changes in the capture antigen [29]. Preliminary experimental results examining the kinetics of ricin-receptor interactions in the presence of JIZ-B7 are not incompatible with the possibility that JIZ-B7 exerts an allosteric effect on ricin, possibly capturing the toxin in a slightly altered conformation that affects the structure of nearby epitopes (A. Poon and N. Mantis, *unpublished results*).

The Tier II V_H_Hs were also effective competitors of the Tier I V_H_Hs (range 79–89%), suggesting (in general) that the Tier I and Tier II epitopes are in close proximity to each other. The JIZ-B7 profile was interesting as it was a good competitor of V5D1 and a weak competitor against the other three Tier II V_H_Hs (V2D4, V4A1, and V8D12), suggesting that V5D1 and JIZ-B7 have considerable epitope overlap. V5D1 was also inhibited by V5E1 (Table 1), which further aids us in locating V5D1’s epitope relative to the other Supercluster II antibodies. Finally, the interaction between the Tier I and Tier III V_H_Hs revealed a wide range of competition profiles, ranging from moderate (68%) to effectively null (6%). We will interpret these profiles later when proposing the relative localization of the Tier III antibody epitopes.

### 2.4. Differential Reactivity with RCA-1 Facilitates Epitope Localization

**Figure 3:**
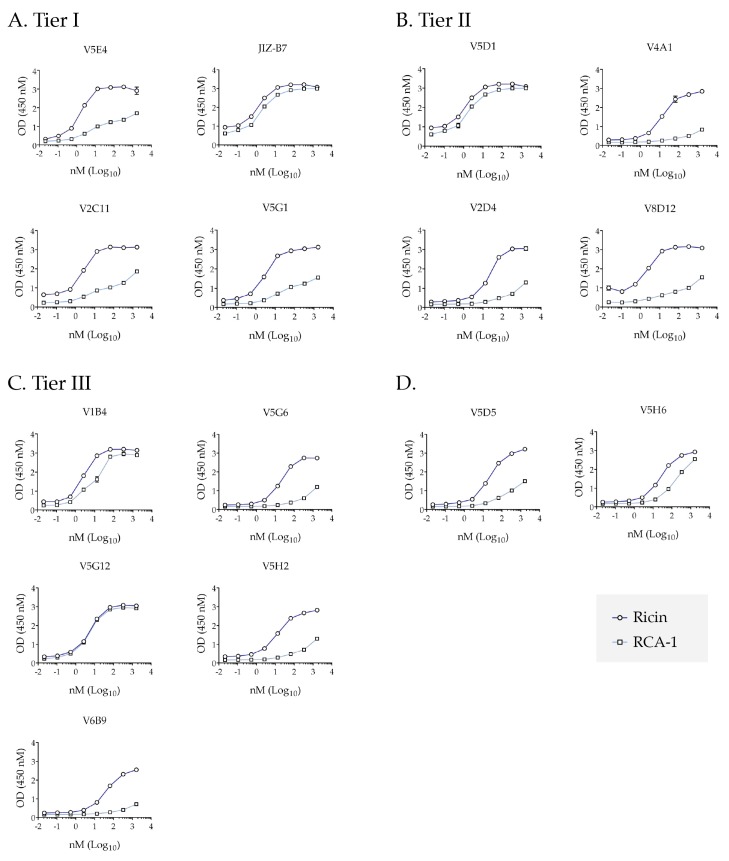
Reactivity profiles of Supercluster II V_H_Hs with ricin and RCA-1. Ricin (circles) and RCA-1 (squares) were captured on microtiter plates using a mAb against RTB’s domain 1 and then probed with indicated Tier I-III V_H_Hs (Panels (**A**–**C**), respectively) or two control V_H_Hs (Panel (**D**)) followed by anti-E-tag-HRP secondary antibody. All V_H_Hs were tested and repeated at least three times.

**Figure 4:**
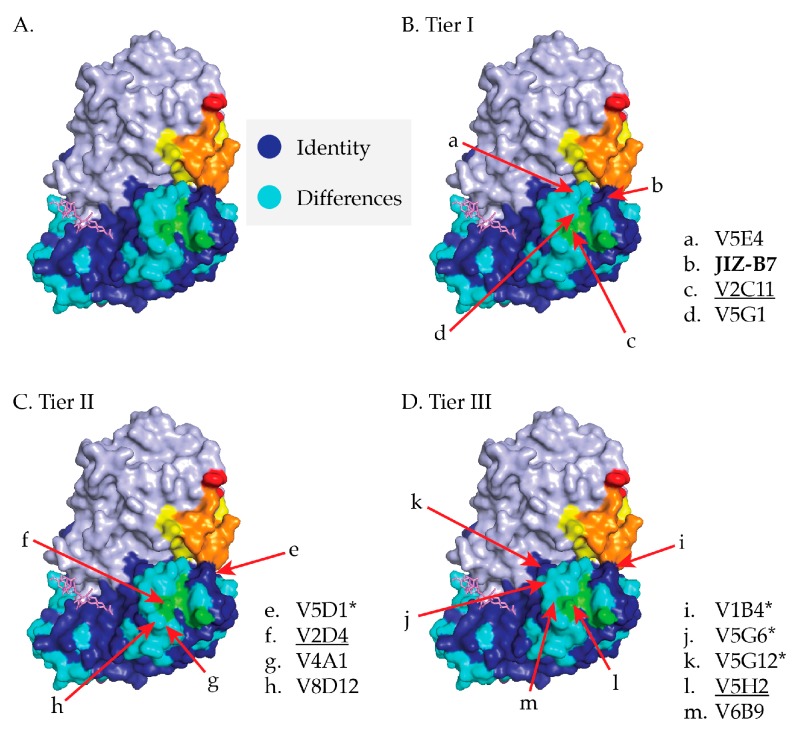
Supercluster II V_H_H epitope positioning on RTB. (**A**) PyMol image of ricin holotoxin with the following landmarks highlighted: RTA (light blue), RTB’s 2γ Gal/GalNAc binding pocket (green), SyH7’s epitope (yellow), V5E1’s epitope (red), and the overlap of SyH7 and V5E1 epitopes (orange). Residues on RTB that are identical on RCA-1 are colored dark blue, while non-identical residues are colored aqua blue. (**B**–**D**) Proposed locations of epitopes recognized by V_H_Hs in Tiers I-III. JIZ-B7 is bolded for emphasis. Asterisks denote holotoxin-specific V_H_Hs, while members of the V2C11 family are underlined.

**Figure 5:**
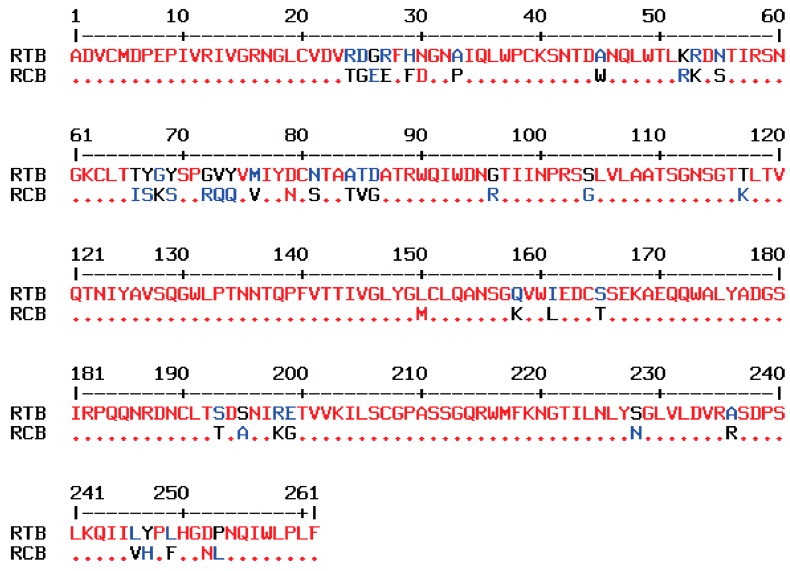
Amino acid sequence alignment of the binding subunits of ricin (RTB) and RCA-I (RCB). Red dots represent regions of sequence identity. Blue amino acids represent regions of low consensus value (less than 50%) and black amino acids represent neutral sequences.

In a separate study, we have shown preliminarily that Tier I and II V_H_Hs recognize recombinant non-glycosylated RTB by ELISA, indicating that the antibodies recognize protein epitopes not RTB’s high mannose side chains (D. Vance, *unpublished results*). Therefore, as an additional strategy for epitope mapping within the supercluster II footprint, we examined the reactivity of the Tier I-III V_H_Hs with *Ricinius communis* agglutinin 1 (RCA-1) (Figure 3) [20]. The B chain of RCA-1 shares 84% amino acid identity with RTB. Within RTB domain 2, in particular, there are well delineated islands of identity and dissimilarity (Figure 4A and Figure 5). Thus, ricin or RCA-1 was immobilized onto microtiter plates and was then probed with the Tier I-III V_H_Hs across a range of concentrations (0.2–2000 nM). Within the Tier I V_H_Hs, JIZ-B7 reacted equally well with RCA-1 and ricin, whereas the other three V_H_Hs (V5E4, V2C11, and V5G1) preferentially reacted with ricin (Figure 3A). Thus, JIZ-B7 recognizes an epitope that is conserved between the toxin and the agglutinin, whereas V5E4, V2C11, and V5G1 recognize ricin-specific epitopes. A similar pattern was observed within the Tier II V_H_Hs in that V5D1 reacted equally well with RCA-1 and ricin, whereas the other three V_H_Hs (V2D4, V4A1, and V8D12) preferentially reacted with ricin (Figure 3B). Finally, the Tier III V_H_Hs displayed a range of reactivity profiles, which will be interpreted in the following section. It is important to note that while RCA-1 profiling experiments are approximate and qualitative in nature, they have proven extremely valuable in relative epitope positioning [20,27,30,31].

### 2.5. Relative Epitope Positioning within Supercluster II

Based on V_H_H competition results and RCA-1 reactivity profiles, we tentatively positioned the epitopes of the 13 Tier I-III V_H_Hs on RTB’s subdomain 2 (Figure 4). The localization studies also took into account previous competition profiles with asialofetuin (ASF) [17], which we have noted when appropriate.

Tier I: Based on the results presented in Table 1 and Figure 3, JIZ-B7’s binding site was localized to a patch of identity on RTB’s subdomain 2γ (238–240) that abuts V5E1’s epitope on RTA and the Gal/GalNAc binding site on RTB. Based on Figure 3 and Table 2, the epitopes recognized by the remaining Tier I V_H_Hs (V5E4, V2C11, V5G1) were positioned along a ridge of dissimilarity that forms a crescent shape around the Gal/GalNAc binding pocket (249–254). V2C11 was placed closer to the 2γ binding pocket based on a reduced binding activity when RTB is complexed with ASF, as described [17]. The two V2C11 family members, V2D4 (Tier II) and V5H2 (Tier III), were assigned the same epitope position as V2C11, based on homology modeling studies conducted with other V_H_H family members [32].

Tier II: V2D4, V4A1, and V8D12’s epitopes were positioned within a region of dissimilarity just below the RTB 2γ Gal/GalNAc binding pocket. The V_H_Hs were positioned further from the RTA interface, as compared to the Tier I V_H_Hs, since they were competed less by V5E1 and the Tier I V_H_Hs themselves. Finally, the epitope recognized by V5D1, a holotoxin-specific antibody, was localized to region close to both JIZ-B7’s putative binding site and V5E1’s epitope on RTA, as defined by X-ray crystallography [24].

Tier III: Tier III V_H_Hs were positioned around the periphery of the Gal/GalNAc binding pocket and at a distance from V5E1’s epitope, with the exception of V1B4, which was shown to compete with SyH7 and V5E1 and demonstrated a near similar reactivity with ricin and RCA-1. V5G6 and V5G12 are holotoxin binders and are therefore positioned close to the interface of RTA and RTB, away from V5E1 and SyH7’s epitope. Lastly, V6B9’s competition profile with Tier I V_H_Hs and its ricin specific reactivity placed its binding site just outside of Tier I V_H_Hs’ epitopes.

### 2.6. TNA Does Not Correlate with V_H_Hs’ Ability to Block Ricin Attachment to Receptors

**Figure 6:**
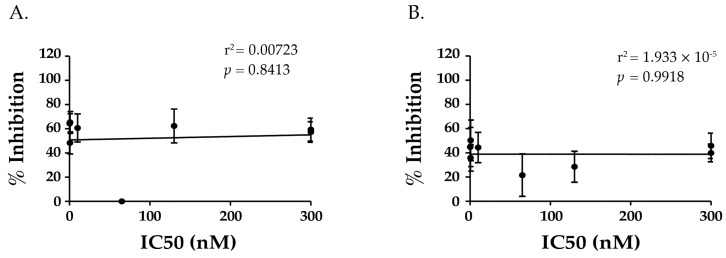
Toxin neutralizing activity of Supercluster II antibodies does not correlate with inhibition of cell attachment. The IC_50_s of the Supercluster II V_H_Hs (from Table 2) and their abilities to inhibit ricin attachment to (**A**) asialofetuin (ASF) and (**B**) THP-1 cells (from Table 3) were subjected to linear regression analysis, as described in the Materials and Methods. There was no correlation in either assay between a V_H_H’s ability to inhibit ricin attachment to receptors and toxin-neutralizing activity.

It is intriguing that most of the V_H_Hs that are characterized in this study are proposed to have binding sites in very close proximity to RTB’s Gal/GalNAc binding pocket, which is known to play a role in the attachment and entry of ricin into host cells [13,33]. It was therefore imperative that we examine what impact, if any, that the V_H_Hs have on RTB-receptor interactions, and determine whether there was a correlation with TNA. We used both cell-based and cell-free assays to test this experimentally. In the cell-based assay, V_H_Hs were mixed with FITC-labeled ricin and then applied to THP-1 cells, a human monocyte cell line, maintained at 4 °C to permit attachment but not toxin internalization and then examined by flow cytometry. In the cell free assay, biotinylated ricin and individual V_H_Hs were mixed together and then applied to microtiter plates that were coated with ASF. ASF is routinely used as a receptor for RTB because it displays three Asn-linked triantennary complex carbohydrate chains with terminal Gal and GalNac moieties [15,33,34]. The results of these assays are presented in Table 3. In essence, there was no correlation between a V_H_H’s IC_50_ and inhibition of ricin attachment to THP-1 cells (Pearson correlation coefficient = −0.004, *p* = 0.9918) or ASF (Pearson correlation coefficient = 0.085, *p* = 0.8413) (Figure 6). In fact, V5D5 is a potent inhibitor of ricin attachment but lacks TNA and is postulated to recognize an epitope outside of Supercluster II (Table 3). JIZ-B7, in contrast, has potent TNA but only affected ricin binding to target cells by ~30%. These results support a model in which the Tier I and Tier II supercluster II antibodies neutralize ricin by a mechanism other than the inhibition of attachment, most likely through the interference with ricin retrograde transport from the plasma membrane to the TGN [35,36]. Further speculation of supercluster II antibodies mechanism of action will be described in the discussion.

## 3. Conclusions and Discussion

**Figure 7:**
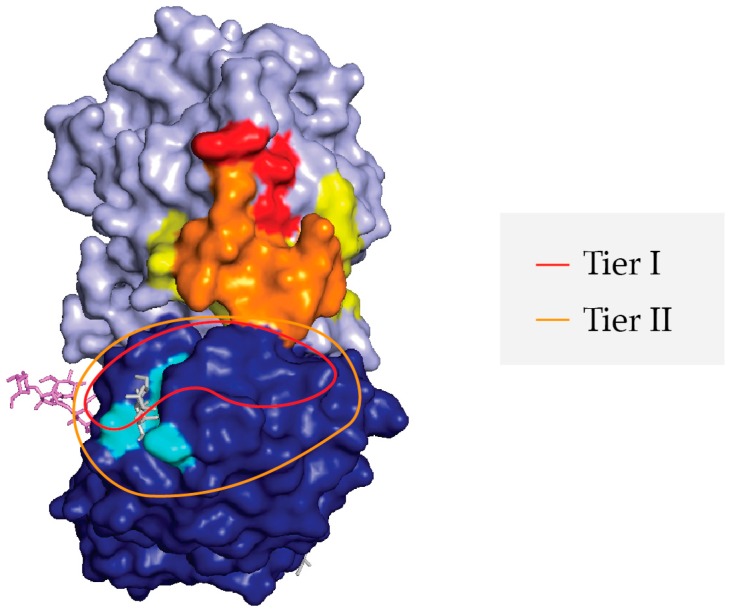
Proposed Supercluster II toxin-neutralizing hotspot at the RTA-RTB interface. PyMol image of ricin holotoxin (as per Figure 1) with the following landmarks highlighted: RTA (light blue), RTB (dark blue), RTB’s 2γ Gal/GalNAc binding pocket (aqua blue), SyH7’s epitope (yellow), V5E1’s epitope (red), and the overlap of SyH7 and V5E1 epitopes (orange). The Tier I V_H_H epitopes are proposed to be confined within the red trace, while the Tier II V_H_H epitopes are proposed to be within the orange trace.

We have tentatively identified an aggregate of B cell epitopes, which we refer to as “supercluster II”, which are located at the RTA-RTB interface. SCII is roughly delineated by SyH7’s binding site on RTA and the Gal/GalNAc CRD on RTB domain 2 (Figure 7). At the core of SCII is a toxin-neutralizing hotspot that is defined by competition ELISAs that includes V5E1 and four RTB-specific Tier I V_H_Hs, JIZ-B7, V5E4, V2C11, and V5G1. The recently available X-ray crystal structure of V5E1 in complex with RTA affords insight into the structural nature of this neutralizing hotspot [24]. V5E1 forms a total of 10 hydrogen bonds and two salt bridges with RTA; contact with loop F–G (residues 192–196) is proposed to be particularly important for neutralizing activity. However, V5E1 also interacts with RTB, as was evident when the RTA-V5E1 complex was superimposed on the structure of ricin holotoxin (Figure 2A) [24]. More specifically, V5E1’s CDR3 likely forms an H-bond with RTB residue Ala237, resulting in 237 Å^2^ of buried surface area. The degree of competition (~95%) between V5E1 and JIZ-B7 for binding to ricin toxin, as shown in Table 1, suggests that the two antibodies engage the same (or closely spaced residues) on RTB. This information was taken into account when positioning JIZ-B7’s epitope on RTB to a patch of RCA-1 amino acid identity located just below V5E1’s binding site on RTA (Figure 4). The epitopes recognized by the remaining three Tier I V_H_Hs (V5E4, V2C11, and V5G1), as well as the Tier II V_H_Hs, were positioned concentrically away from V5E1’s contact point based on values obtained in competition ELISAs (Figure 4 and Figure 6). Within the Tier I and II V_H_Hs, toxin-neutralizing activity was associated with proximity to V5E1’s epitope, not the RTB’s Gal/GalNAc CRD, further supporting the notion of there being a core neutralizing hotspot that is situated at the RTA-RTB interface. That said, we recognize that the competition ELISAs and differential reactivity profiles with RCA-1 presented in this manuscript enable us to only approximate the location of the epitopes that are recognized by Tier I-III V_H_Hs. More definitive epitope positioning awaits the structural analysis of a Tier I V_H_Hs in complex with ricin holotoxin, as well as mutagenesis of residues on ricin toxin proposed to be involved in antibody contact. Both these tracks of investigation are currently ongoing.

RTB should be an “easy target” for neutralizing antibodies, yet very few have been identified, and of those that have been described, their mechanisms of action remain incompletely defined. As a rule, antibodies against RTB have been proposed to function by one of two mechanisms: blocking ricin attachment to cell surfaces (so-called type I) or interrupting ricin intracellular trafficking (so called type II) [36,37]. The results presented in Table 3 and Figure 6 argue against the Tier I V_H_Hs (JIZ-B7, V5E4, V2C11, and V5G1) interfering with RTB’s ability to engage with host cell receptors. Therefore, we speculate that JIZ-B7, as well as V5E4, V2C11, and V5G1, likely influence toxin endocytosis and/or retrograde transport. This would be consistent with the proposed mode of action of cluster II antibodies like SyH7, which delayed ricin's egress from EEA-1(+) and Rab7(+) vesicles, and enhanced toxin accumulation in LAMP-1(+) vesicles, suggesting a degradation in lysosomes [37]. Going forward, it will be extremely interesting to compare the effects of V5E1 and JIZ-B7 on ricin endocytosis, retrograde transport, and even the efficiency of RTA retrotranslocation. Legler and colleagues demonstrated recently that RTA-specific V_H_Hs with toxin-neutralizing activity limit the RTA thermal unfolding and/or conformational changes that would likely be encountered during toxin endocytosis (e.g., low pH). It is possible that the Tier I and II V_H_Hs described in our current study may similarly influence the stability of RTB. However, when compared to RTA, very little is known about RTB’s unfolding dynamics. Even the issue of cooperativity between RTB’s two carbohydrate recognition domains remains unresolved [38]. We have only just initiated experiments to define the influence of pH on RTB association/dissociation with Gal/GalNAc residues and how single chain antibodies like JIZ-B7 might influence those dynamics.

On a final note, it is interesting to speculate that “SCII-like” mAbs may have been first identified several decades ago. For example, Chanh and colleagues described a toxin-neutralizing mAb BG11-G2, which did not bind to either purified ricin chain A or chain B, but recognized an antigenic determinant whose conformation requires the combination of the two chains [39]. Others have also described holotoxin-specific mAbs with toxin-neutralizing activity [40,41]. While there are likely many quaternary B cell epitopes along the RTA-RTB interface, it is clear from our current study that supercluster II is a region of the toxin that is vulnerable to neutralization.

## 4. Methods

### 4.1. Chemicals, Biological Reagents and Cell Lines

Labeled and unlabeled ricin toxin (*Ricinus communis* agglutinin II) and *Ricinus communis* agglutinin I (RCA-I) were purchased from Vector Laboratories (Burlingame, CA, USA). Ricin was dialyzed against phosphate buffered saline (PBS) at 4 °C in 10,000 MW cutoff Slide-A-Lyzer dialysis cassettes (ThermoFisher Pierce Protein Biology, Rockford, IL, USA) prior to use in cytotoxicity studies. Unless noted otherwise, all of the other chemicals were obtained from Sigma-Aldrich (St. Louis, MO, USA). Vero cells and THP-1 cells were obtained from the American Type Culture Collection (ATCC, Manassas, VA, USA), and propagated as recommended by ATCC. Cell culture media were provided by the Wadsworth Center’s tissue culture facility.

### 4.2. V_H_Hs and mAbs

Expression and purification of the ricin-specific single domain antibodies (V_H_H) was done as described previously [16,24,25]. The murine mAb SyH7, as described previously [22], was purified from hybridoma supernatants by Protein A chromatography at the Dana Farber Cancer Institute’s Monoclonal Antibody Core facility (DFCI, Boston, MA, USA).

### 4.3. ELISA

Epitope profiling immunocompetition capture ELISAs (“EPICC”) were performed as follows: Nunc Maxisorb F96 microtiter plates (ThermoFisher Scientific, Pittsburgh, PA, USA) were coated overnight with 1 µg/mL capture mAb (SyH7) or V_H_Hs (V5E1, V5E4, JIZ-B7, V2C11, or V5G1). Plates were blocked with 2% goat serum, washed, and incubated with analyte V_H_Hs (330 nM) that had been mixed with biotinylated-ricin (b-R). The amount of b-R that was used in the competition ELISA was adjusted to achieve the EC_90_ of each capture antibody. After 1 h, the plates were washed and developed with streptavidin-HRP antibody (1:500; SouthernBiotech, Birmingham, AL, USA) and 3,3′,5,5′-tetramethylbenzidine (TMB; Kirkegaard & Perry Labs, Gaithersburg, MD, USA). The plates were analyzed with a SpectraMax 250 spectrophotometer equipped with Softmax Pro 7 software (Molecular Devices, Sunnyvale, CA, USA). The percent (%) inhibition of ricin binding to capture antibody in the presence of competitor V_H_H was calculated from the optical density (OD) values as follows: 1-value OD_450_ (biotin-ricin + analyte V_H_H)/value OD_450_ (biotin-ricin without analyte V_H_H) × 100. For RCA-1 ELISAs, microtiter pates were coated with 24B11 (1 μg/mL) for 1 h and then incubated with either ricin or RCA-1 (1 μg/mL) for 1 h and then blocked overnight. The query V_H_Hs were serially diluted (1:5), starting at 1.65 μM and detected using goat anti-E tag-HRP secondary antibody [16].

### 4.4. Vero Cell Cytotoxicity Assays

Vero cell cytotoxicity assays were performed as previously described [42]. Vero cells were detached from culture dishes with trypsin, adjusted to ~5 × 10^4^ cells per ml, and seeded (100 µL/well) into white 96-well plates (Corning Life Sciences, Corning, NY, USA), and allowed to adhere overnight. Cells were then treated with ricin (0.01 µg/mL; 154 pM), ricin:V_H_H mixtures, or medium alone (negative control) for 2 h at 37 °C. Cells were then washed and incubated with 10% FBS in medium for 48 h. Cell viability was assessed using CellTiter-GLO (Promega, Madison, WI, USA). All of the treatments were performed in triplicate, and 100% viability was defined as the average value obtained from wells in which cells were treated with medium only. All of the experiments were repeated at least three times independently.

### 4.5. THP-1 Cell Attachment Assay

THP-1 cell attachment assays were performed as previously described [20]. THP-1 cells were collected by gentle, low speed centrifugation (400× *g* for 5 min), adjusted to 5 × 10^6^ cells per mL and seeded (200 µL/well) into clear U-bottom 96-well plates (BD Bioscience, San Jose, CA, USA). Cells were kept on ice at 4 °C for 20 min before treatment and throughout the experiment to prevent toxin internalization. Cells were then incubated with either FITC-labeled ricin (200 ng/well), FITC-ricin:V_H_H (330 nM) or with serum-free medium as a negative control for 30 min. Cells were then washed with ice cold PHEM buffer three times to remove unbound V_H_H:ricin complexes and were fixed in 4% formaldehyde in PHEM. Fluorescence was measured using the FACS Calibur flow cytometer (BD Bioscience, San Jose, CA, USA). A minimum of 10,000 events was analyzed per sample; experiments were repeated three independent times.

### 4.6. Statistical Analyses

Statistical analyses were performed in GraphPad Prism version 7.03 (GraphPad Software, San Diego, CA, USA). The relationship between IC_50_ values and percent inhibition of Tier I and II V_H_Hs, as described in Figure 2, was done using linear regression analysis. Tier III V_H_Hs were excluded due to the lack of a quantifiable IC_50_ value. To compare the levels of V5E1 competition of individual V_H_Hs, as shown in Table 2, we performed a one-way ANOVA with Dunnett’s multiple comparisons test. *p* values < 0.05 were considered significant.

### 4.7. Modeling of Ricin Toxin

Images of ricin holotoxin (PBD ID 2AAI) or RTA-V_H_H complexes were generated using PyMOL (The PyMOL Molecular Graphics System, Schrodinger LLC, San Diego, CA, USA).

## Figures and Tables

**Table 1:** Classification and characterization of SCII V_H_Hs.

Competitive Binding Assays						
Tier ^a^	V_H_H	Target	K_D_^b^ [nM]	TNA ^c^ (IC_50_)	V5E1 ^d–f^	Bin ^g^
I	V5E4	RTB	0.006	0.3	87 ± 12	3
	**JIZ-B7**	RTB	0.0306	0.6	95 ± 2	4
	V2C11	RTB	0.0154	0.8	88 ± 12	4
	V5G1	RTB	0.106	5	86 ± 16	5
II	V5D1	Holotoxin	0.216	65	85 ± 5	1
	V2D4	RTB	3.2	130	64 ± 17 *	6
	V4A1	RTB	0.666	300	62 ± 16 *	6
	V8D12	RTB	0.194	300	62 ± 10 *	n.a.
III	V1B4	Holotoxin	4.09	-	85 ± 7	2
	V5G6	Holotoxin	1.24	-	37 ± 6 **	7
	V5G12	Holotoxin	0.993	-	42 ± 12 **	2
	V5H2	RTB	2.17	-	38 ± 3 **	7
	V6B9	RTB	1.61	-	40 ± 14 **	n.a.
Control	V5D5	RTB	5.54	-	7 ± 12 **	10
	V5H6	RTB	18.3	-	9 ±16 **	8

^a^ V_H_Hs are grouped based on TNA (IC_50_): Tier I: IC_50_ < 10 nM; Tier II: IC_50_ 10–300 nM; Tier III: IC_50_ > 300 nm; ^b^ Binding affinities (nM) were determined by SPR and reported in a separate study [17]; ^c^ TNA was determined in a Vero cell cytotoxicity assay and repeated at least three times; ^d,e^ V5E1 competition was determined using biotinylated ricin competition assays, as described in the Materials and Methods. V5E1 competition values represented in % inhibition; ^f^ One-way ANOVA was performed on V5E1 competition (% inhibition) and was followed up with a Dunnet Test, producing *p*-values, comparing V5E1 vs. itself (control). * *p*-value < 0.05; ** *p*-value < 0.0001. JIZ-B7 is bolded for emphasis. The V2C11 family is underlined; ^g^ Bins are competition profiles based on mAb competitions as adapted from [17].

**Table 2:** Epitope localization of SCII V_H_Hs by competition ELISA.

Capture V_H_Hs					
Tier	Analyte ^a^	V5E1	JIZ-B7	V2C11	V5G1
I	V5E4	96 ± 2 ^b^	48 ± 11	96 ± 1	95 ± 2
	**JIZ-B7**	96 ± 1	96 ± 1	96 ± 2	95 ± 2
	V2C11	96 ± 2	42 ± 9	95 ± 3	95 ± 2
	V5G1	94 ± 3	43 ± 4	94 ± 3	94 ± 3
II	V5D1 *	80 ± 10	82 ± 8	79 ± 12	82 ± 12
	V2D4	83 ± 1	35 ± 11	82 ± 4	89 ± 2
	V4A1	83 ± 3	29 ± 8	88 ± 1	88 ± 2
	V8D12	84 ± 16	31 ± 10	89 ± 8	88 ± 10
III	V1B4 *	43 ± 12	45 ± 4	40 ± 11	70 ± 6
	V5G6 *	22 ± 2	10 ± 2	29 ± 7	44 ± 11
	V5G12 *	9 ± 3	13 ± 1	6 ± 5	29 ± 14
	V5H2	47 ± 5	16 ± 6	53 ± 3	67 ± 7
	V6B9	68 ± 4	27 ± 7	68 ± 13	79 ± 4
Controls	V5D5	2 ± 2	2 ± 1	3 ± 2	19 ± 14
	V5H2	2 ± 3	1 ± 2	4 ± 5	10

^a^ Indicated V_H_Hs (column) were mixed in solution with biotinylated ricin (80 ng/mL) and applied to microtiter plates coated with indicated capture V_H_Hs (top row). ^b^ Values represent % inhibition of ricin binding ± standard deviation from at least 3 replicates. The asterisks (*) denote V_H_Hs whose epitopes are holotoxin-specific. JIZ-B7 is bolded for emphasis. Underlines indicate members of the V2C11 family of V_H_Hs.

**Table 3:** V_H_H interference of ricin attachment to host cell receptors.

% Inhibition			
Tier	V_H_H	ASF ^a^	THP-1 ^b^
I	V5E4	64 ± 8	45 ± 16
	**JIZ-B7**	48 ± 9	36 ± 11
	V2C11	65 ± 11	50 ± 16
	V5G1	60 ±12	44 ± 12
II	V5D1 *	0	22 ± 17
	V2D4	62 ± 14	28 ± 12
	V4A1	59 ± 9	40 ± 7
	V8D12	57 ± 8	46 ± 10
III	V1B4 *	19 ± 18	27 ± 18
	V5G6 *	38 ± 13	16 ± 14
	V5G12 *	0	3 ± 14
	V5H2	55 ± 2	40 ± 6
	V6B9	57 ± 3	38 ± 8
Controls	V5D5	84 ± 10	90 ± 1
	V5H6	6 ± 11	18 ± 8

Binding assays were performed as described in Materials and Methods. Listed V_H_Hs were ^a^ premixed with biotinylated ricin (0.25 µg/mL) and was applied to ASF coated plates or ^b^ premixed with FITC-labeled ricin (1 μg/mL), applied to THP-1 cells, and subjected to flow cytometry. Numbers listed represent % ricin binding inhibition ± standard deviation for three replicates. The asterisks (*) denote V_H_Hs whose epitopes are holotoxin-specific. JIZ-B7 is bolded for emphasis. Underlines indicate members of the V2C11 family of V_H_Hs.

## Supplementary Materials

The following are available online at www.mdpi.com/2072-6651/9/12/378/s1, Table S1: VHH-SyH7 Competition Results, Figure S1: Sequence alignments of four Tier I VHHs and V2C11 family VHHs.
