# Supplementary material for: A Supercluster of Neutralizing Epitopes at the Interface of Ricin’s Enzymatic (RTA) and Binding (RTB) Subunits
Source: Toxins (Basel). 2017 Nov 23;9(12):378. doi: 10.3390/toxins9120378 (PMC5744098; doi:10.3390/toxins9120378)
Supplement: Supplementary file 1 [file toxins-09-00378-s001.pdf]

Supplementary Materials: A Supercluster of Neutralizing Epitopes at the Interface of Ricin's Enzymatic (RTA) and Binding (RTB) Subunits

Amanda Y. Poon, David J. Vance, Yinghui Rong, Dylan Ehrbar, and Nicholas J. Mantis

Table S1. VHH-SyH7 Competition Results.

Tier	V_HH	% Inhibition	
		Capture[a]	EPICC[b]
I	V5E4	85 ± 0.5	48 ± 0.9
	RTB-B7	71 ± 0.3	96 ± 0.1
	V2C11	88 ± 0.1	54 ± 8.1
	V5G1	88 ± 0.3	53 ± 0.8
II	V5D1*	75 ± 1.5	89 ± 1.9
	V2D4	92 ± 0.5	34 ± 12.9
	V4A1	92 ± 0.4	29 ± 6.1
	V8D12	ND[c]	45 ± 9
III	V1B4*	90 ± 0.4	86 ± 5
	V5G6*	86 ± 0.5	28 ± 1
	V5G12*	88 ±0.7	34 ± 11
	V5H2	88 ± 1.1	20 ± 1.3
	V6B9	ND	16 ± 2.6
Controls	V5D5	7	8 ± 8.9
	V5H6	22 ± 4.1	7 ± 1

[a] % inhibition values determined by sandwich ELISA in which SyH7 was used as a capture antibody, adopted from Vance et al., (2017 Clin Vaccine Immunol. 10.1128/CVI.00236-17). [b] Competition between SyH7 and VHHs determined using biotin-ricin capture assay, as described in Materials and Methods. [c] ND, not determined. * indicates V_HHs that are holotoxin-specific (i.e., recognize ricin toxin, but not either of the individual subunits, RTA or RTB).

A.

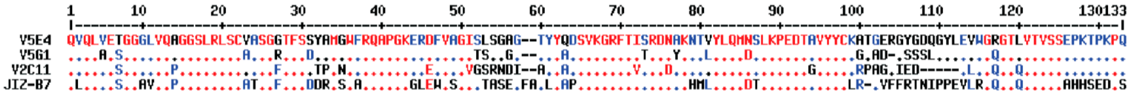

B.

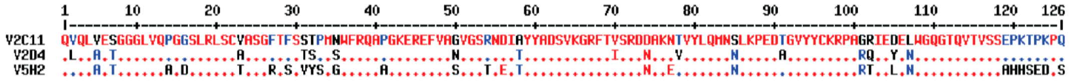

Figure S1. Sequence alignments of four Tier I VHHs and V2C11 family VHHs. Amino acid sequence alignment of the (**A**) Tier I VHHs and (**B**) V2C11 family. Red dots represent identical sequences and blue dots represent amino acids that are the same as the first sequence in the alignment. Red amino acids represent regions of high consensus, blue amino acids represent regions of low consensus and black amino acids are neutral. Dashes mean no amino acid in that position.
